# More Carrots, Less Sticks: Encouraging Good Stewardship in the Global Antimicrobial Commons

**DOI:** 10.1007/s10728-023-00455-x

**Published:** 2023-02-13

**Authors:** Cristian Timmermann

**Affiliations:** Ethics of Medicine, Medical Faculty, University of Augsburg, Augsburg, Germany

**Keywords:** Common-pool resources, Global justice, Poverty sensitive, Antibiotic

## Abstract

Time-tested commons characterize by having instituted sanctioning mechanisms that are sensitive to the circumstances and motivations of non-compliers. As a proposed Global Antimicrobial Commons cannot cost-effectively develop sanctioning mechanisms that are consistently sensitive to the circumstances of the global poor, I suggest concentrating on establishing a wider set of incentives that encourages both compliance and participation.

Looking back at Elinor Ostrom’s ground-breaking analysis of common-pool resources, we find that when it comes to punishment fellow commons members frequently looked into the motivations of rule-breakers and their excuses [11]. Efforts were made to continuously ensure that members learn about the rules and what is expected from them. Many long-standing commons have also paid attention that the different members do not face too harsh struggles to comply with the rules. Here mechanisms were developed to be softer on first-time offenders and significant efforts were made to reason with rule-breakers to gain their compliance. It seems that members of commons widely embrace an old principle from modern philosophy that has fallen into oblivion: the right to necessity [9]. Necessity justifies a commensurable breaking of rules if doing so allows us to meet basic needs that could not have been met otherwise. In traditional commons, coercive systems were sensitive to the needs and circumstances of its members.

Moving onwards to present-day global commons proposals, such as the Global Antimicrobial Commons [4], the international community will face major hurdles when establishing coercive measures that are sensitive to the circumstances of its different subjects. Top-down approaches will likely fail, as there is a widespread epistemic ignorance among technocrats to the circumstances of poorer countries. Even within countries, political authorities have lost touch to the problems faced by the average citizen. As a result, coercive measures will often be too soft on some and too hard on others, jeopardizing the social cohesion that needs to be in place to maintain a well-functioning commons. More democratic solutions will likely lead to better decision-ownership but too modest measures to slow down significantly antibiotic resistance.

To design good rules, we need to start with the assumption that people who want to assemble or join a commons are willing to comply with reasonable measures. Scholarship on distributive justice and retributive justice place too much emphasis on players in a levelled field who do not want to play by the rules, and not enough on working with people of different backgrounds and capacities who want to play by the rules but will occasionally fail to do so. Contributive justice, on the other hand, is much more oriented towards empowering people to do their share to support the common good [18]. An appeal to promote the common good is a central motivation to collectively understand a good as a commons and to join forces to establish such a commons [19]. This is why time-tested commons spend a considerable effort teaching their members the rules and build sufficient social cohesion to secure cooperation. To maintain a sense of community it is essential that members retain their ability to play by the rules, which requires adaptations and relaxations in times of individual distress.

A Global Antimicrobial Commons should encourage people endowed with vastly different resources to adhere to a basic rule: use antibiotics only when truly needed. To evaluate whether antibiotics are necessary, within a collective, each potential user needs to ask herself what is lost when not undergoing an antibiotic treatment. Assessing someone’s decision to seek antibiotics requires substantial knowledge on their circumstances. First, people need a minimum of health literacy to not look for antibiotics for every kind of health complication. As long as people are convinced that antibiotics will help them they will find someone who is willing to sell them [1]. False conceptions of “necessities” need to be eradicated, otherwise people will think that they are wrongfully denied access to a resource they have a legitimate claim on, reducing trust towards the health care system and the government. Second, social security allows people to postpone treatments and makes it less risky and costly to do so. The absence, or inadequacy, of social security provision, particularly paid sick leave, and labour protection laws shifts all risks towards the individual considering treatment. Rules will not be seen as fair and erode social cohesion when it is so much more difficult for some to comply with them in contrast to others [17]. Health workers are also likelier to overprescribe when patients cannot risk longer absences from work and therefore request a rapid solution [5, 2]. Third, countries and regions have different health burdens [10]. As disease burden increases the number of cases recommending the use antibiotics, it also likelier that misapplications occur. As people everywhere benefit from antibiotic stewardship, a fair sharing of costs also needs to consider that higher disease burdens make antibiotic disease monitoring more difficult [7, 13].

The idea that states should be free to individually specify their commitments is a major step [16] in recognizing differential capacities. Yet one of the major problems within the Global South is the inability to secure long-term political commitment. Political parties who come to power often take an opposite approach than their predecessors and many undo their opponents’ progressive policies for the sole sake of discrediting them. Harsh sanctions and ‘legal hooks’ may disincentivize state officials from making stronger commitments, as they are well aware of political volatilities and may want to avoid future accusations against them.

As it is enormously costly for a Global Commons to establish adequate sanctioning capacities that are sensitive to the needs of its poorer members, we need to prioritize the development of adequate incentives and leave to a second stage the role of punishment.

To establish a Global Antimicrobial Commons, Rogers Van Katwyk and colleagues [15] are right to point out the historic responsibility richer countries have in contributing to antibiotic resistance. These countries are also well endowed to enforce measures that will act as strong incentives to implement policies to extend the active life of antibiotics that will ultimately benefit all. Yet here it is important to adopt another principle of time-tested commons: to allow polycentric governance [12]. The establishment of new incentives should ideally give birth to new centres of governance that allow enthusiastic newcomers to take a leading role in the fulfilment of specific tasks within the commons and make best use of their local knowledge.

To start, people need to become aware and gain confidence that by acting together they can prolong the effective life of this vital resource and thereby address a fundamental interest of people in the present and the future [6]. This requires a basic understanding of both collective action problems and opportunities. Common members need to inform each other and potential members about both the harms resulting from misled aggregate actions and the multiple benefits of cooperating in securing appropriate access, coordinating conservation efforts and joining forces in innovation. Common members can benefit from all advantages of cooperation, such as pooling risks, sharing information, making mutual commitments, and engaging in projects of multiple scales [3]. Newcomers who want to assume a leading role can act as regional coordinators of public awareness campaigns on the benefits of cooperation and the need to tackle together antibiotic resistance.

Second, there is ample evidence that disease burden negatively affects GDP [14] and that investments in promoting healthier lifestyles have enormous returns [8]. Commons members need to share information and experiences about measures that are relatively cheap to implement and about the fact that improving antibiotic stewardship also helps reduce costs in the short-term. Members who already adopted conservation measures can share with newcomers their experiences and local adaptations.

Third, as most of the poorer countries are highly indebted, progress towards improving health infrastructure, with special emphasis on preventing antibiotic resistance, needs to be acknowledged and seen as valuable assets that increase future economic capacity when renegotiating debts. The Global North can use its influence to make sure such improvements are duly acknowledged when it comes to assessing risks for credit worthiness and debts repayments.

Fourth, lax labour laws and increasingly precarious employment conditions lead to an increase in demand for antibiotics, often even against medical indication in the hope to speed up recovery [1]. The COVID-19 pandemic has made clear again that people who fear employment sanctions for taking sick leave or cannot financially afford to miss work are becoming a major impediment to control infectious diseases. Punishing people who transmit diseases, or overuse antibiotics to speed up recovery, does not lead to compliance when people cannot secure subsistence needs through other means than work. It is therefore imperative that labour protection rights are re-implemented and expanded. Countries of the Global North need to pressure their corporations to be the examples of good practice and not be the main drivers of deregulation by tax-exempting lobbying activities.

Fifth, to facilitate innovation, countries of the Global North should finally make stronger concessions by making transparency on the source of biological resources mandatory in patent applications and so encourage biodiversity rich countries to join efforts in a broader search for new antibiotic compounds. As researchers from biodiversity rich countries gain confidence that they will get a fair reward and recognition for the biological resources they share with Commons members, they can establish regional innovation centres and offer guidance to emerging research institutions in similar circumstances.

A Global Commons that disproportionately overburdens some of its members is prone to erode as it cannot maintain a sufficient level of social cohesion. In contrast to other treaties, a Global Antimicrobial Commons requires establishing adequate incentives rather than strict policing. Yet here the COVID-19 pandemic is providing a policy window by making it clear that the existing health systems are a serious liability and that urgent reforms of global scope are needed.

## Data Availability

Not applicable.
